# Interplay between MRI radiomics and immune gene expression signatures in oral squamous cell carcinoma

**DOI:** 10.1038/s41598-025-96821-x

**Published:** 2025-04-12

**Authors:** Anna Corti, Deborah Lenoci, Valentina D. A. Corino, Davide Mattavelli, Marco Ravanelli, Tito Poli, Stefano Cavalieri, Lisa Licitra, Loris De Cecco, Luca Mainardi

**Affiliations:** 1 Department of Electronics, Information and Bioengineering, Politecnico di Milano, Via Ponzio 34/5, Milan, 20133 Italy; 2 Integrated Biology of Rare Tumors, Department of Research, Fondazione IRCCS, Istituto Nazionale dei Tumori, Milan, Italy; 3 Cardiotech Lab, Centro Cardiologico Monzino IRCCS, Milan, Italy; 4 Unit of Otorhinolaryngology-Head and Neck Surgery, Department of Medical and Surgical Specialties, Radiological Sciences, and Public Health, ASST Spedali Civili of Brescia, University of Brescia, Brescia, Italy; 5 Unit of Radiology, Department of Medical and Surgical Specialties, Radiological Sciences, and Public Health, ASST Spedali Civili of Brescia, University of Brescia, Brescia, Italy; 6 Maxillo-Facial Surgery Division, Head and Neck Department, University Hospital of Parma, Parma, Italy; 7 Head and Neck Medical Oncology Department, Fondazione IRCCS Istituto Nazionale dei Tumori, Milan, Italy; 8 Department of Oncology and Hemato-Oncology, University of Milan, Milan, Italy

**Keywords:** Magnetic resonance imaging, Radiomic features, Transcriptomic signatures, Head and neck cancer, Tumor microenvironment, Radiogenomic, Cluster analysis, Biomarkers, Cancer, Cancer genomics, Cancer imaging, Oral cancer, Tumour biomarkers, Cancer, Scientific data, Statistics

## Abstract

With the advances in immunotherapy and the challenge of poor responsiveness in oral squamous cell carcinoma (OSCC) patients, understanding the tumor microenvironment is crucial. Radiogenomics offers the potential to provide pre-operative, non-invasive image-derived immune biomarkers. To this aim, the present study explores the capability of MRI-based radiomics to describe patients’ immune state in OSCC. Seven MRI-based radiomic, 29 immune-related gene expression signatures were computed and deconvolution analysis was performed for a subset of OSCC from the BD2Decide database. A correlation-driven analysis identified key associations between radiomic and immune-related signatures and cell populations. Radiomic classifiers of the gene expression signatures were then developed to evaluate their capability to stratify patients based on immune status. MRI-based radiomic models showed promising results in predicting a gene expression signature associated with significant prognostic value for HNSCC patients who underwent radiotherapy (AUC = 0.92), suggesting these models’ potential in distinguishing radioresistant from radiosensitive patients, aiding treatment decisions. Additionally, radiomic signatures reflected immune infiltrating cells in our cohort (M1, CD8 + T, B cells). MRI-radiomic signatures and associated models could become non-invasive methods to evaluate the prognosis and treatment choice in OSCC patients. Based on our promising results, and upon external validation, MRI-radiomics could enhance personalized medicine approaches.

**Supplementary Information:**

The online version contains supplementary material available at 10.1038/s41598-025-96821-x.

## Introduction

Oral squamous cell carcinoma (OSCC) is one of the most common types of head and neck malignancies, and, together with lip cancer, accounted for 389,485 incident cases and 188,230 deaths in 2022 worldwide^1^. Current treatment strategies for head and neck squamous cell carcinoma (HNSCC), including OSCC, mainly encompass surgery, chemotherapy and radiotherapy. With the understanding of the crucial role that the immune system plays in the formation, development, and progression of HNSCC^2,3^, immunotherapy emerged as a promising cure in HNSCC and recently received increased interest^4^. Despite the recent advances and the exciting results obtained from the treatment with immune checkpoint inhibitors, the response rate to immunotherapy is still low^5^. In this context, deciphering the complex immune landscape of HNSCC is crucial for understanding the reason for differential responses to immunotherapy and for identifying potential immunotherapeutic targets^6,7^. To this aim, a large number of studies were proposed leading to the development of immune-related gene expression signatures reflecting tumor diversity and potentially related to survival or immunotherapy response^8^. However, gene expression analysis requires tissue biopsies, that can be biased by tumor heterogeneous composition, while the whole tumor tissue is available only after surgical excision, thus precluding the use of immunotherapy upfront. Thus, although transcriptomic analysis and gene expression biomarkers have significantly contributed to a better tumor characterization, the localized nature of the analysis prevents from obtaining comprehensive pre-surgical information about patient-specific immune tumor processes.

Radiomics, namely the quantitative extraction and mining of high-throughput features from radiological images, has emerged as a potential source of non-invasive image biomarkers of tumor heterogeneity, prognosis and treatment response and has been largely investigated in HNSCC^9,10^. Moreover, radiogenomics, namely the association of radiomic features extracted from pre-operative radiological images and transcriptomic data obtained from tumor biopsies holds the potential to provide pre-operative, non-invasive image-derived immune biomarkers, as demonstrated by recent radiogenomic studies related to immune processes in HNSCC^11–18^. The underlying hypothesis is that tumor biological pathways would also be reflected in the radiographic images, and thus in the radiomic features. These studies focused on the development of radiomic signatures from computer tomography images for the prediction of the expression levels of specific genes, as granzyme A, CD27 or PDL1, or for the prediction of gene expression signatures defining CD8 + T-cell enrichment. However, to the best of the authors’ knowledge, radiogenomic studies in HNSCC based on radiomic features extracted from magnetic resonance imaging (MRI) are lacking, although MRI is often the modality of choice in HNSCC patients. Moreover, while previous studies explored the potentiality of radiomic features to predict the expression of single genes or specific gene signatures related to immune processes in HNSCC, a wider analysis encompassing a large number of published immune-related gene expression signatures in HNSCC has not been proposed yet. Finally, all the aforementioned studies considered HNSCC, without focusing on a specific tumor sub-site (i.e., pharynx, hypopharynx, oral cavity and lip, larynx, ), although HNSCC represents an extremely heterogeneous class of tumors. Considering all the above, radiogenomic studies focused on immune-related processes based on MRI radiomics and specific for OSCC are lacking.

In this scenario, the aim of the present study is to provide a comprehensive radiogenomic analysis to explore the capability of MRI-based radiomics to describe patients’ immune state in OSCC. To this aim we considered a subset of OSCC from the BD2Decide database for which radiomics and transcriptomics data were available. First, literature-derived MRI radiomic features and gene expression signatures were selected; second, reproducible MRI-based radiomic signatures and immune-related gene expression signatures specific for HNSCC were reproduced on our OSCC dataset; third, a correlation-driven cluster analysis was performed to detect key associations between radiomic and immune-related signatures and cell populations; and finally, radiomic classifiers of the gene expression signatures were developed to evaluate the capability of a single radiomic signature or their combination to stratify patients based on their immune status.

## Results

**Fig. 1:**
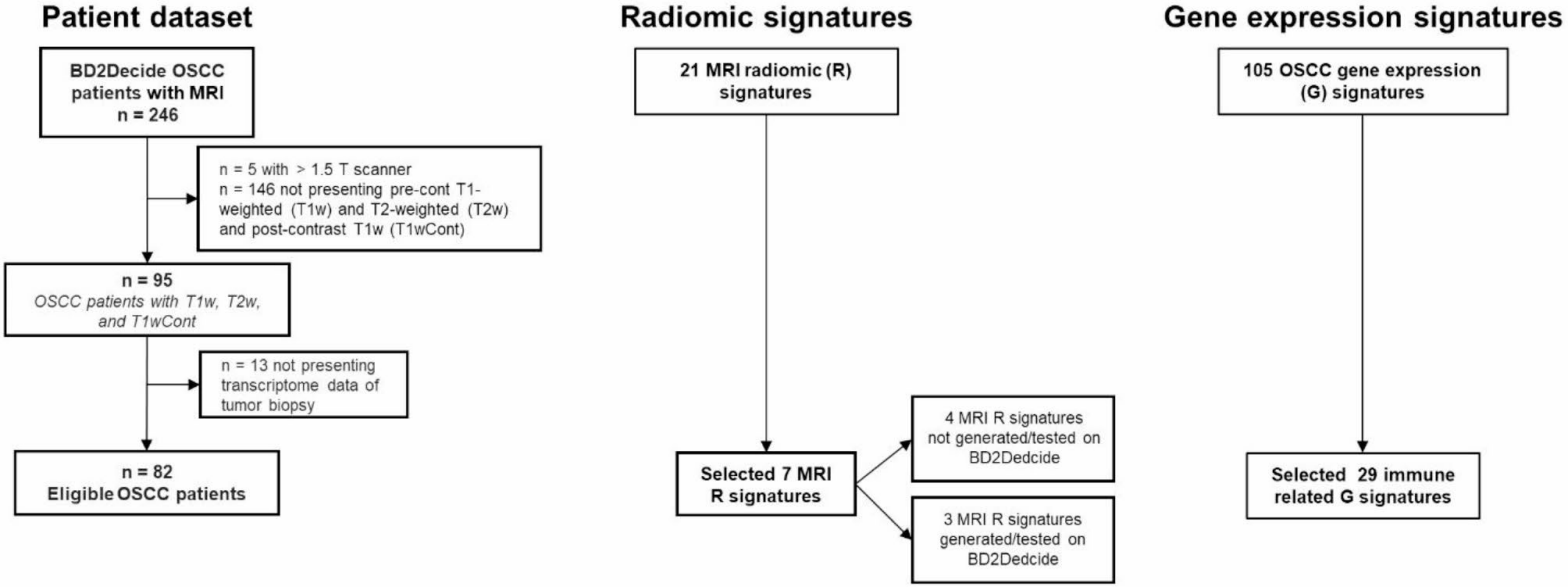
Consort.

Figure 1 provides the consort of the patient dataset, the radiomic signatures and the gene expression signatures, further detailed in the following sections.

### Patient characteristics

Table 1 summarizes the baseline clinical information of a subset of the BD2Decide database^19^, i.e. the 82 patients affected by stage III/IVa-b OSCC included in the study according to the availability of radiomic features and transcriptome data to be analyzed.

**Table 1:** Clinical data of the patients used for the study.

	Dataset *N* = 82
Date of diagnosis	2008–2017
Median follow-up	27.91 months (IQR 17.86–44.28)
Gender	
M	43 (52%)
F	39 (48%)
Median age	63 y (IQR 55–72)
cTNM 8th edition	
III	17 (21%)
IVa/b	65 (79%)
Smoking status	
Current/former	43 (52%)
Never	26 (32%)
Unknown	13 (16%)

### Radiomic signatures

From the literature survey, 21 papers were initially found and analyzed to identify the ones reporting the list of features and coefficients, needed to reproduce the radiomic signature. Seven reproducible MRI-based radiomic signatures for HNSCC patients were identified^20–26^ and reproduced on the OSCC dataset (Table 2).

Five monomodal signatures were reported: three of them based on features extracted from the post-contrast T1-weighted (T1wCont) sequence (R1, R2 and R4) and the remaining ones from the pre-contrast T2-weighted (T2w) sequence (R5 and R7). R3 and R6 are multimodal signatures, with R3 based on pre-contrast T1-weighted (T1w), T1wCont and T2w sequences and R6 on T1w and T2w sequences. Overall, 34 constituent features (21 from T1wCont, 12 from T2w and 1 from T1w) emerged, with one feature selected in both R6 and R7 (Supplementary Table 1 details, for each radiomic signature, the considered radiomic features).

The 7 radiomic signatures were computed on our OSCC dataset of 82 patients, by following the methods detailed in the studies and reported in the methods section^27^.

**Table 2:** Reproducible MRI-based HNSCC radiomic prognostic signatures.

Radiomic signature	HNSCC datasets (*N*° samples)	*N*° of features	Image modality
R1 *Bos 2021* ^20^	Oropharynx (177)	10	T1wCont
R2 *Chen 2022* ^21^	Hypopharynx (190)	6	T1wCont
R3 *Alfieri 2022* ^22^	HETeCo (57) and BD2Decide (137) – HNSCC (oral cavity, oropharynx, larynx, hypopharynx)	3	T1w, T1wCont, T2w
R4 *Siow 2022* ^23^	hypopharynx (198)	4	T1wCont
R5 *Mossinelli 2023* ^24^	Oral cavity cancer (79)	2	T2w
R6 *Bologna 2023* ^25^	BD2Decide (519) – HNSCC (oral cavity, oropharynx, larynx, hypopharynx)	5	T1w, T2w
R7 *Corti 2023* ^26^	BD2Decide (231) – oral cavity	5	T2w

### Gene expression signatures

Based on already present literature evidences of immune status relevance in HNSCC^2,3^, we identified from the literature survey 29 gene expression signatures related to the immune pathway. Their main characteristics are reported in Table 3 and Supplementary Table 2.

**Table 3:** Immune-related gene expression signatures: main characteristics.

Gene expression signatures	HNSCC datasets (*N*° samples)	Number of Genes	Deconvolution algorithm
G1 *Li L*,*2020* ^28^	all subsites: TCGA (501)	21	TIMER
G2 *Fang R*,* 2021* ^29^	all subsites: TCGA (416); oral cavity cancer: GSE41613 (96)	10	CIBERSORT
G3 *Li S*,*2021* ^30^	oral cavity cancer: TCGA, ICGC, GSE41613, GSE42743, GSE75538, (317, 40, 97, 71, 14)	11	CIBERSORT
G4 *Lin X*,* 2021* ^31^	oral cavity cancer: TCGA, GSE41613 (314, 97)	8	MULTIGSEA
G5 *Bai S*,* 2019* ^32^	oral cavity cancer: TCGA, GSE41613 (314, 97)	18	Not done
G6 *Lv S*,* 2022* ^33^	oral cavity cancer: TCGA, GSE41613 (314, 97) IMvigor210 (348)	8	CIBERSORT
G7 *Feng J*,* 2021* ^34^	oral cavity cancer: TCGA (328)	11	CIBERSORT
G8 *Dai D*,* 2021* ^35^	all subsites locally advanced: TCGA, GSE65858 (288,270)	31	xCell, quantiseq, TIMER, MCP-counter, epic
G9 *Wang Z*,* 2021* ^36^	all subsites: TCGA, GSE65858 (500, 270)	6	TIMER
G10 *QiangW*,* 2021* ^37^	all subsites: TCGA, GSE65858 (500, 270)	13	CIBERSORT
G11 *Chen J*,* 2022* ^38^	all subsites: TCGA, GSE65858 (500, 270) oral cavity cancer: ICGC (28)	12	CIBERSORT
G12 *Yang J*,* 2020* ^39^	all subsites: TCGA (499); oral cavity cancer: GSE41613 (97)	6	CIBERSORT
G13 *Long J*,* 2020* ^40^	all subsites: TCGA (499)	14	TIMER
G14 *LiangY*,* 2019* ^41^	all subsites: TCGA (504)	11	Not done
G15 *Chen N*,* 2022* ^42^	all subsites: TCGA, E-MTAB-8588 (499, 99)	6	CIBERSORT
G16 *Li J*,* 2022* ^43^	all subsites: TCGA (500) oral cavity cancer: GSE41613 (97)	7	TIMER
G17 *Zhang Y*,* 2021* ^44^	all subsites: TCGA (504)	3	Immune Identifier
G18 *Ming R*,* 2021* ^45^	all subsites: TCGA GSE65858 (499, 270) oral cavity cancer: GSE41613 (97)	13	CIBERSORT
G19 *Zhang Y*,* 2022* ^46^	all subsites: TCGA (501) GSE176221 (9)	3	CIBERSORT
G20 *Yi L*,* 2020* ^47^	all subsites: TCGA GSE65858 (499, 270)	15	CIBERSORT
G21 *Feng B*,* 2020* ^48^	all subsites: TCGA, GSE65858 (502, 270)	10	CIBERSORT
G22 *Chen Y*,* 2021* ^49^	all subsites: TCGA, GSE65858 (502, 270)	3	CIBERSORT
G23 *Ming R*,* 2022* ^50^	all subsites: TCGA (499) oral cavity cancer: GSE41613 (97)	15	CIBERSORT
G24 *Chen Y*,* 2021* ^51^	all subsites: TCGA, GSE65858 (500, 270)	9	CIBERSORT
G25 *Lu Y*,* 2022* ^52^	all subsites: TCGA (499) oral cavity cancer: GSE41613 (97)	7	TIMER
G26 *Cai Z*,* 2022* ^53^	all subsites: scRNA-seq GSE139324 (26) TCGA (490)	9	Not done
G27 *Zhang Y*,* 2021* ^54^	all subsites: TCGA, GSE65858 (500, 270)	6	TIMER
G28 *Tang X*,* 2022* ^55^	all subsites: TCGA, GSE102349 (494, 88)	14	CIBERSORT
G29 Zhang S, 2022 ^56^	all subsites: TCGA (498) oral cavity cancer: scRNA-seq GSE103322 (18) 8	8	CIBERSORT

*Algorithm applied for immune cells identification in the original study: CIBERSORT: 16; TIMER: 6; Immune Identifier: 1; not done: 3; xCell: 1; MultiGSEA:1.

### Radiomics correlates with immune gene expression signatures

**Fig. 2:**
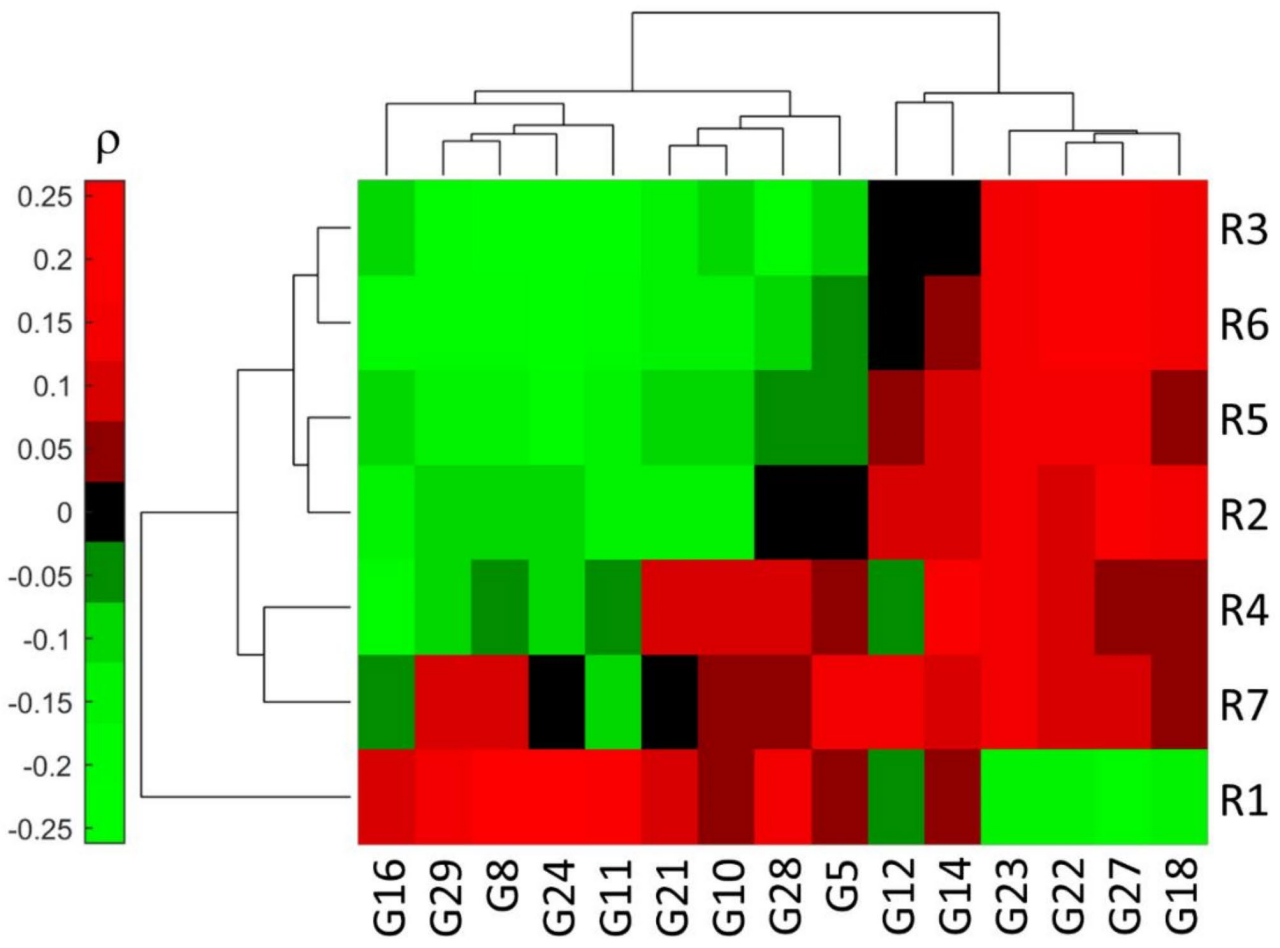
Correlation-driven clustering and dendrogram between the 7 radiomic (R) signatures and the selected 15 gene expression (G) signatures. Only stable clusters were depicted. Spearman’s correlation coefficient (ρ) was computed between each R and G signature. The main characteristics of R and G signatures are respectively reported in Tables 2 and 3.

After performing the silhouette analysis for the identification of stable correlation-based clusters, 15 out of the initial 29 immune gene expression signatures were retained. Figure 2 shows the correlation-driven clustering and dendogram between each radiomic signature and immune-related signature, resulting in 4 clusters presenting direct and inverse relationships with the radiomic signatures. Specifically, 5 gene expression signatures (G16, G29, G8, G24, G11) were characterized by a positive correlation with R1 and a negative correlation with R2, R3, R4, R5 and R6; 3 gene expression signatures (G10, G28, G5) presented a positive correlation with R1, R4 and R7, and a negative correlation with R5 and R6; the G14 signature presented a positive correlation with all the radiomic signatures, except for R3; 4 gene expression signatures (G23, G22, G27 and G18) were characterized by a negative correlation with R1 and a positive correlation with all the other radiomic signatures.

**Fig. 3:**
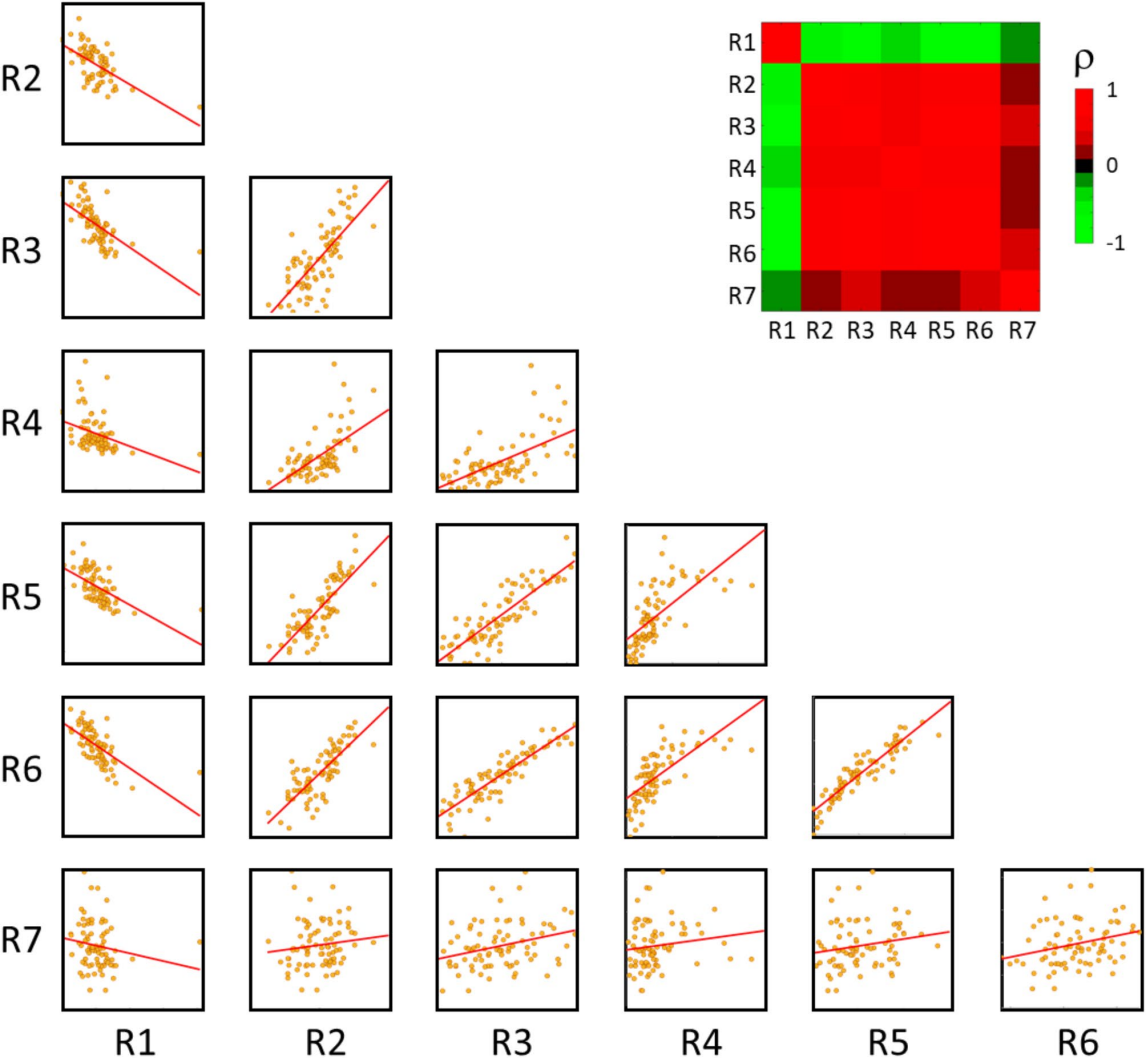
Paired scatter plots between the 7 radiomic signatures (R1-R7) and correlation matrix heatmap of Spearman’s correlation coefficient (ρ).

**Fig. 4:**
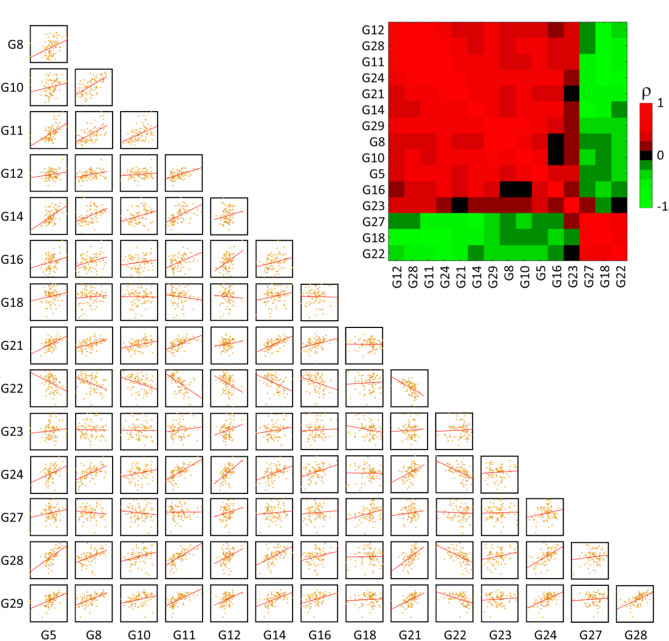
Paired scatter plots between the 15 gene expression (G) signatures and correlation matrix heatmap of Spearman’s correlation coefficient (ρ).

The identified radiomic-gene expression relationships align with the internal correlations observed among the radiomic and gene expression signatures. Specifically, as depicted in Fig. 3 for the radiomic signatures, R1 demonstrated a negative correlation with all other signatures, while the remaining radiomic signatures exhibited positive correlations with each other. This consistency reflects the methodology behind the development of these radiomic signatures: R1 was designed such that higher values were associated with improved prognosis^20^, whereas the opposite was designed for all other signatures^20–26^. Regarding the gene expression signatures, G27, G18 and G22 scarcely positively correlated with G23 and were inversely correlated with all the remaining gene expression signatures (Fig. 4).

### Radiomic models predict radiosensitivity-related gene expression

**Fig. 5:**
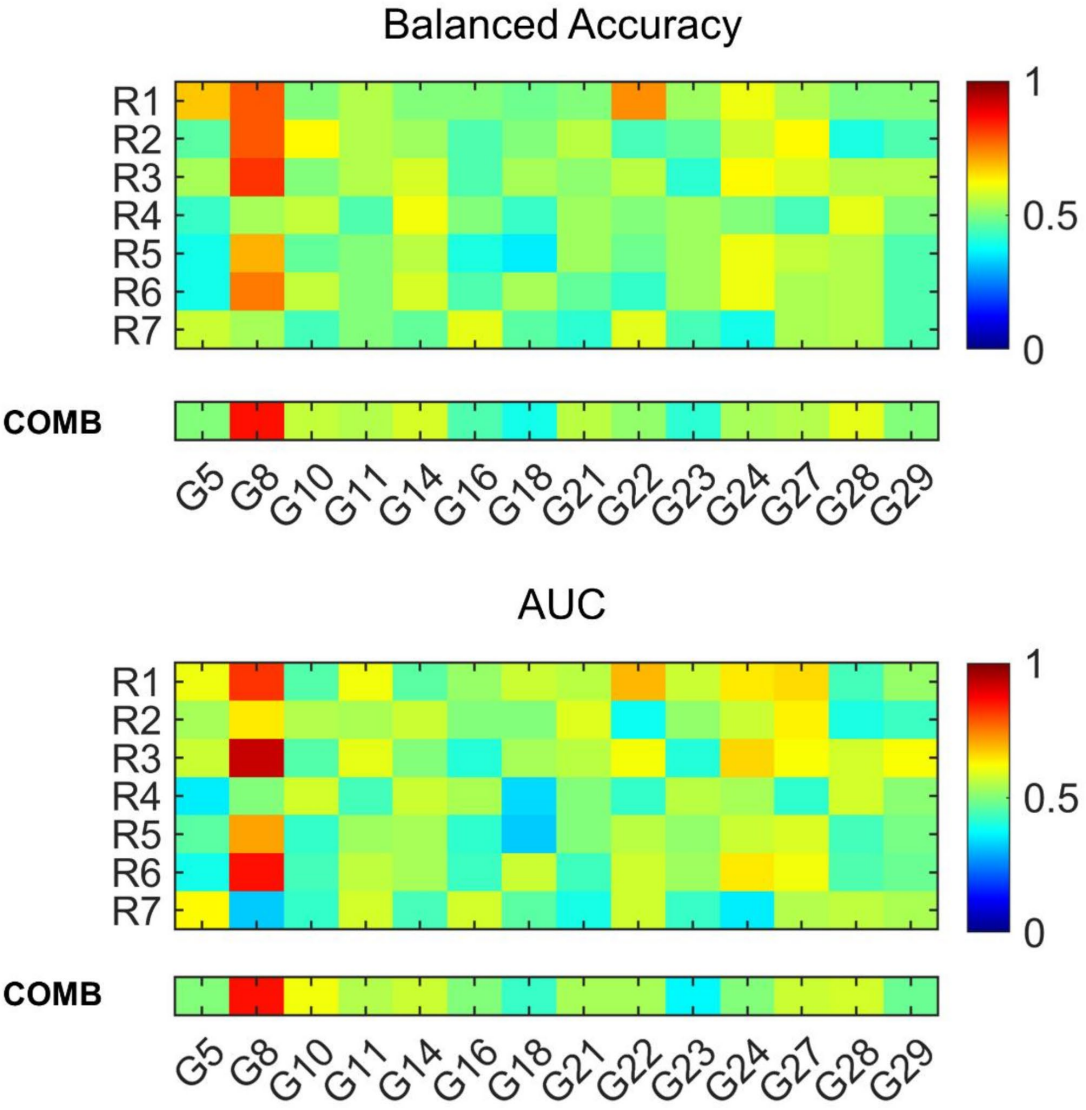
Median balanced accuracy and area under the receiver operating characteristic curve (AUC) over 100 repetitions of the single radiomic models (top) and combined radiomic model (bottom) of each gene expression signature included in the study.

Radiomic predictive models were built for the identified highly correlated immune-related gene expression signatures that presented a minority class greater than 10% of the dataset. Accordingly, G12, was excluded from the analysis. Radiomic predictive models of the gene expression signatures were developed considering each radiomic signature alone and the combination of all radiomic signatures. Figures 5 shows the median balanced accuracy and area under the receiver operating characteristic curve (AUC) over 100 repetitions for the single and the combined radiomic models. Overall, the best performances were obtained for the prediction of G8^35^, which, as detailed in the Supplementary Table 2, was based on the combination of 31 radiosensitivity-related genes and PD-L1 expression, and was developed for the prediction of prognosis in patients with locally advanced HNSCC.

Details about the median and IQR balanced accuracy and AUC of the radiomic models of G8 are provided in Table 4. Specifically, 3 over 7 radiomic signatures (R1, R3 and R6) stratified patients according to G8 expression with a median balanced accuracy  > 0.75 and a median AUC > 0.82. R1, R3 and R6 were associated with median balanced accuracy of 0.79, 0.82 and 0.75 and median AUC of 0.82, 0.93 and 0.86. The combination of radiomic signatures provided a median balanced accuracy and AUC of 0.86. Overall, the combined radiomic model was associated with a higher balanced accuracy compared to all single radiomic models, and a higher AUC compared to all single radiomic models but R3 and R6.

**Table 4:** Performance metrics of the radiomic models of gene expression signature G8, as median (interquartile range).

	R1	R2	R3	R4	R5	R6	R7	Comb
**B acc**	**0.79** **(0.75 0.86)**	0.79 (0.37 0.82)	**0.82** **(0.39 0.86)**	0.54 (0.14 0.59)	0.70 (0.61 0.75)	**0.75** **(0.68 0.79)**	0.54 (0.25 0.57)	**0.86** **(0.39 0.89)**
**AUC**	**0.82** **(0.68 0.93)**	0.64 (0.43 0.93)	**0.93** **(0.57 1.00)**	0.50 (0.21 0.79)	0.71 (0.50 0.93)	**0.86** **(0.57 0.93)**	0.32 (0.14 0.50)	**0.86** **(0.50 0.93)**

In bold: radiomic models with median balanced accuracy (B acc) > 0.75 and median area under the receiver operating characteristic curve (AUC) > 0.82.

### Radiomics reflects immune cell populations infiltration

**Fig. 6:**
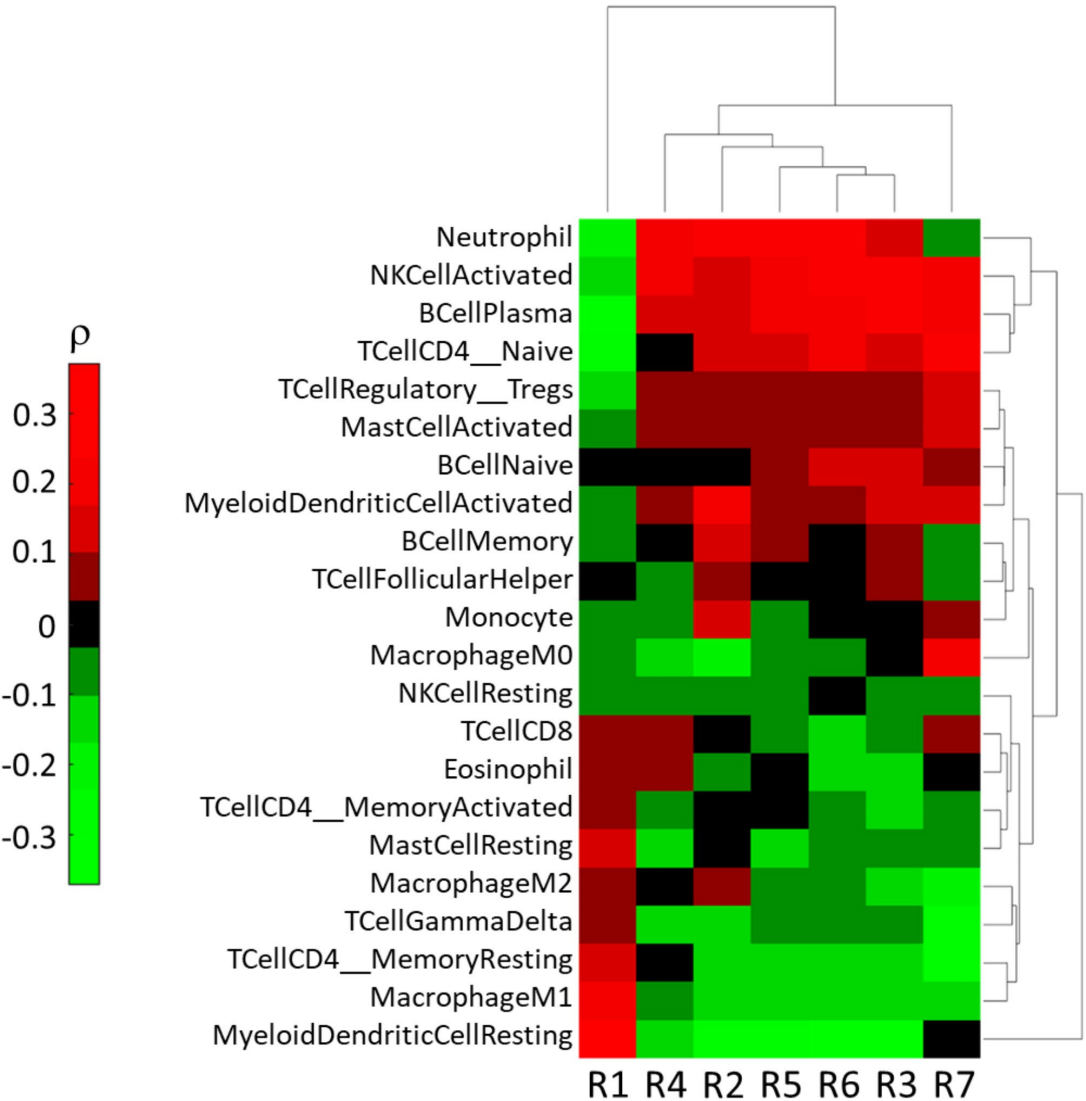
Correlation between radiomic signatures (R1-R7) and immune populations identified by Cibersort in the 82 OSCC patients. Spearman’s correlation coefficient (ρ) was computed.

The correlation between radiomic signatures and immune cell population on our cohort is reported in Fig. 6. Based on the results of the radiomic predictive model of G8 obtained with R1, R3 and R6 (as shown in Table 4), and the evidence that R1 was negative correlated to all the other signatures presented in Fig. 3, only the immune cells correlated to R1 and R3 /R6 were summarized in Table 5.

**Table 5:** Correlation of radiomic signatures R1, R3 and R6 with immune cells present in the tumor microenvironment of our patient dataset.

Correlation of radiomic signatures with immune cells*	
R1	R3 and R6
Positive	
(+++) = Myeloid dendritic resting, Macrophage M1 (++) = T cell CD4 memory resting, Mast cell; (+) = Tcell CD8, Eosinophil, T cell CD4 Memory activated, Macrophage M2, T cell Gamma Delta	(++) = Neutrophil, NK activated, B cell, Bcell naïve, T cell CD4 naïve (+) = Myeloid Dendritic Cell Activated
Negative	
(--) = B cell, T cell CD4 naïve, Neutrophil, Tcell regulatory Tregs	(--) = T cell CD8, Eosinophil, T cell Gamma Delta, Macrophage M1, Myeloid dendritic resting,

* level evaluated by Cibersort in the tumor micoenvironment of OSCC 82 patients.

## Discussion

With the advent and progresses of immunotherapy and the unsolved issue related to scarce responsiveness in HNSCC patients, understanding the tumor microenvironment has become of utmost importance. Herein, by comprehensively exploring the association between 7 MRI-based radiomic (based on T1w, T1w-Cont and T2w image modalities) and 29 immune-related gene expression signatures on a well characterized OSCC clinical dataset, we obtained promising results highlighting the presence of key associations between radiomics and OSCC patients’ immune state. By correlation-based cluster analysis, radiomic models predicting high/low expression of 15 gene expression signatures were developed by considering both single radiomic signatures and their combination.

In particular, MRI-based radiomic models showed promising results in predicting gene expression signature G8, particularly with R1, R3 or R6 radiomic signatures or when the combination of the 7 radiomic signatures was considered. Among the 29 signatures analyzed, based on a different number of genes (range 3–31; mean and median 10) the G8^35^ was: (i) based on the highest number of genes, i.e. 31 radiosensitivity-related genes (ACTN1, ANXA2, ANXA5, ARHGDIB, CAPNS1, CBR1, CCND1, CD63, CORO1A, CXCR4, DAG1, EMP2, HCLS1, HTRA1, ITGB5, LAPTM5, LRMP, MYB, PFN2, PIR, PKM, PTMS, PTPRC, PTPRCAP, PYGB, RAB13, RALB, SCRN1, SQSTM1, TWF1, WAS); and (ii) associated with a significant prognostic value for HNSCC patients who underwent radiotherapy^35^, suggesting its potential utility in distinguishing radioresistant from radiosensitive patients and significantly contributing in treatment decisions. Notably, of the 82 OSCC patients analyzed, 50 (61%) underwent radiotherapy, and the radiomic signatures evaluated in the study were designed to predict the prognosis of HNSCC patients. This suggests that the prognostic radiomic signatures may effectively predict a gene expression signature related to radiosensitivity, which contains prognostic information for patients who received radiotherapy. Furthermore, radiotherapy was found to be linked to immune system activation, as immune profiling revealed that radioresistant tumors were less likely to show immune activation, particularly with a decreased presence of B cells^57^. This sheds light on the relationship between immune response, radiosensitivity, and prognosis in HNSCC, potentially explaining the link between the prognostic radiomic signatures and the radiosensitivity-related G8 signature.

Furthermore, radiomic signatures were found to reflect also immune infiltrating cells in our cohort. Among positively correlated immune cells, R1 detected high presence of Macrophages M1, moderate presence of CD8 + T cells, and absence of B cells, while R3 and R6, and most of remaining radiomics signatures, showed an opposite scenario with high presence of B cells and low presence/absence of Macrophage M1 and CD8 + T cells. Macrophages show a dual function in the microenvironment of HNSCC: they can suppress cancer progression (M1) and also play a pro-cancer role promoting immune evasion, invasion, metastasis and angiogenesis (M2)^58^. CD8 + T cells play an important anti-cancer role activating immune response against cancer cells. They can recognize the MHC peptide complexes expressed by cancer cells through T cell receptor (TCR) inducing cancer cells death. B cells exhibit an anti-cancer function by antibody-dependent cell cytotoxicity, complement activation and T cell activation via antigen presentation^59^.

In the tumor microenvironment, the balance between M1 and M2 can be deregulated in favor of the immunosuppressor phenotype, CD8 + T cells could express an exhausted phenotype and B cells can also promote tumor proliferation enhancing inflammation and immunosuppression environment. Generally, the expression of macrophage M1, CD8 + T cells and B cells in human tumors is associated with good prognosis. Moreover, these cells are also an important target in current checkpoint blockade immunotherapy^60^.

The identified correlation of radiomic profile with immune cell infiltration in HNSCC was in line with the study by Sun et al.^18^, and Katsoulakis et al.^13^. However, while these studies specifically focused on identifying CT-based radiomic correlates of T cell CD8, herein a wider analysis was performed, including 22 cell populations, and MRI rather than CT radiomic features were considered.

In the present study we demonstrated the potential utility of providing a radiomic surrogate of the immune-relate gene expression G8, that, with an overall median AUC up to 0.92, and median balanced accuracy up to 0.86, can be used as pre-operative predictor of radiosensitivity. The predictive performance of our radiomic models is in line with that obtained in similar radiogenomic studies focused on immune-related processes in HNSCC, reporting AUC ranging from 0.52 to 0.80 in the validation set^11–18^. However, to the best of the authors’ knowledge, this is the first study that explores radiogenomic associations in HNSCC settings considering validated and publicly available radiomic signatures and immune-related gene expression signatures, rather than single radiomic features and immune-related genes, as previously proposed^11–18^. On this basis, even these results are promising, only after validation with more precise methods (such as single-cell RNA). The here reported MRI-based radiomic signatures could represent a significant advancement as a pre-surgical, non-invasive, and cost-effective alternative to gene expression signatures. At present, since the presence of immune cells in tumor microenvironment was detectable but not yet correctly measured, considering the heterogeneous status of immune cells, their association with radiomic signatures is not yet well defined.

The study was not exempt from limitations. First, the analysis was based on a relatively small cohort of 82 OSCC patients from the BD2Decide dataset, who met the inclusion criteria: larger and prospective studies will be required to validate our findings. Moreover, only seven literature-derived reproducible radiomic signatures prognostic for overall survival in HNSCC were considered in the present study, and some assumptions were introduced to replicate them on our dataset, accounting for the insufficient details reporting (e.g., features normalization for R1 and R5). Third, radiomic and gene expression signatures originally developed for HNSCC across various tumor locations were considered and replicated on OSCC patients. This decision was driven by the availability of fully reproducible radiomic and gene expression signatures in the literature. HNSCC studies provided a robust dataset with well-established radiomic features and immune gene expression signatures, serving as a valuable foundation for our research. It is important to note that the gene expression signatures were derived from The Cancer Genome Atlas (TCGA)^8^, where 62% of HNSCC patients (172 out of 279) had oral cavity tumors, making the dataset highly representative of OSCC and compatible with our dataset. Additionally, radiomic signatures R5 and R7 were specifically designed for OSCC patients, while R3 and R6 were developed for HNSCC patients, with a substantial proportion having oral cavity tumors, further supporting their relevance. For instance, R3 was trained on a dataset where 46% of patients had OSCC and validated on a dataset with 67% OSCC^22^; R6 was trained on a dataset with 47% OSCC and validated on a dataset with 46% OSCC^25^.

The inclusion of radiomic signatures that were not specifically developed for gene expression prediction or tailored to OSCC likely contributed to the relatively modest predictive performance observed. However, despite these limitations, the findings of this study are encouraging. We demonstrated that certain radiomic signatures can indeed predict gene expression profiles, establishing a foundation for future research in this area. Notably, while G8 signature exhibited superior predictive performance, we also observed promising results for other gene expression signatures, with balanced accuracy and AUC values exceeding 0.60. These results suggest there are underlying relationships between radiomic features and gene expression profiles, even though the weaker predictive outcomes may be partly attributed to the limitations of the signatures used. This highlights the need for further research aimed at developing radiomic signatures that are more specifically tailored for OSCC and gene expression prediction, which could enhance predictive power and clinical relevance in future studies. Moreover, the findings underscore the importance of the reproducibility of radiomic analyses to advance the field and enable potential applications in personalized medicine. In this context, our study not only demonstrates the potential of radiomic features in predicting gene expression signatures in OSCC but also illuminates the current gaps in the field. This should encourage future efforts toward transparent, fully reproducible radiomic analyses, and the development of OSCC-specific radiomic and gene expression studies. As more accessible and reproducible radiomic signatures, ideally tailored to OSCC, become available, future research can build upon and expand the work presented here.

Overall, the present study provides remarkable contribution in the emerging field of radiogenomics aimed to identify non-invasive radiomic surrogates of gene expression signatures related to immune processes in HNSCC, being the first study that (i) considers MRI-based radiomics, (ii) explores a large number of immune-related gene expression signatures and (iii) is specific for OSCC. In conclusion, MRI-radiomic signatures and associated models, thanks to effective detection of the immune status of patients, could become non-invasive methods to evaluate the prognosis/treatment choice in OSCC patients. On the basis of our promising results, and upon validation on external cohorts, we envisage that MRI-radiomics could improve personalized medicine approaches.

## Methods

### Analyzed dataset

The study involved a subset of 82 loco-regionally advanced (clinical TNM III/IV according to the 8th edition of AJCC/UICC) OSCC patients of the BD2Decide project (NCT02832102)^19^ already used or partially used for MRI radiomic prognostic signatures generation or validation^22,25,26^. Eighty-two patients with (i) T1w, T2w and T1wCont MRI image sequence (acquired with 1.5 T scanner) and (ii) whole-transcriptome data from tumor tissue were analyzed. Patient data were collected from two participating centers: 68 patients from the Azienda Ospedaliera Universitaria di Parma (Italy) and 14 patients from the Spedali Civili di Brescia (Italy) (as subcontractor of IRCCS Istituto Nazionale dei Tumori of Milan, Italy). The study was approved by the Ethical Committees of the participating centers; data acquisition followed the General Data Protection Regulation of the EU and informed consent was obtained from all patients.

### Radiomic signatures

A literature survey was performed to retrieve reproducible MRI-radiomic prognostic signatures in HNSCC patients and compute the radiomic scores on our OSCC dataset. The minimum requirement to reproduce the signatures was information on the features and corresponding coefficients. The search was conducted by considering studies from 2015 to today within the public database “Pubmed” (www.ncbi.nlm.nih.gov/pubmed) and using keywords as “Head and neck”, “radiomics”, “MRI” and “survival”.

#### Image data acquisition, segmentation

Image data were generated as already described in details in previous studies^22,25,26^. In particular, T1w, T2w and T1wCont MRI were acquired using scanners with a field strength of 1.5 T and a turbo spin-echo pulse sequence. The contouring of the gross tumor volume was performed at the clinical centers using a semi-automatic segmentation software based on coupled shape modeling^61^. The region of interest (ROI), corresponding to the primary tumor, was segmented manually slice by slice by HNSCC expert radiologists (one for each center). T2w sequence was considered as reference to segment the ROI and the other sequences (T1w an T1wCont) were used to check and correct the segmentations.

#### Radiomic signatures computation

To reproduce the literature-derived MRI-radiomic signatures, the following steps were considered, according to the methods declared in the original radiomic studies: first, MRI images were preprocessed, second, radiomic features were extracted and normalized and third, the signatures were computed for each patient as the linear combination of the features and the corresponding coefficients^27,62^. As regards image preprocessing, commonly adopted methods include: (i) denoising, through a 3D Gaussian filter with a 3 × 3 × 3 voxel kernel and σ = 0.5; (ii) intensity non-uniformities correction, through the N4ITK algorithm^63^; (iii) intensity standardization, using Z-score; (iv) voxel size resampling to a specific isotropic resolution, through B-spline interpolation^64^ and (v) fixed-bin histogram discretization, with a specific number of bins. In case the considered studies did not report specific methodologies for image preprocessing, the default settings of Pyradiomics 2.2.0 software (open-source, available at https://github.com/Radiomics/pyradiomics and run on Python, used to extract the radiomic features) were considered: 25-bins histogram discretization^65^.

Radiomic features extraction was performed using Pyradiomics 2.2.0. Radiomic features were extracted from the original image and transformed images, including the Laplacian of Gaussian (σ = 0.5, 1.0, 1.5, 2.0 and 5.0 mm) the wavelet, the square, the square root and the logarithm filters^66^. For each original and transformed image, features belonging to first order statistics, shape and size (only for original images), grey level cooccurrence matrix, grey level size zone matrix, neighboring gray tone difference matrix and grey level dependence matrix were extracted, for a total of *n* = 5064 features. Features were processed according to the methods declared in the original studies. If information regarding features normalization was missing, the original features were considered, while if features normalization was mentioned, but without providing additional details, Z-score normalization was applied on our features^27^.

The identified reproducible radiomic signatures were thus computed for each patient of the dataset as the linear combination of the features and the corresponding regression coefficients.

### Gene expression signatures and deconvolution analysis

A survey of literature was performed to retrieve gene expression signatures from patients with HNSCC/OSCC. The search was conducted by considering studies from 2019 to today using as inclusion criteria “HNSCC or OSCC” AND “immune status”. The identified papers were selected if presented: (i) data obtained by whole gene expression data; (ii) complete description of the bioinformatics methods; (iii) availability of the gene list, weights, and algorithm to compute signature’s score. The genes were re-annotated based on EntrezID^67^ and the bioinformatics methods were retrieved from the original manuscripts to reproduce the signature’s algorithms allowing applying them on external datasets along with thresholds for patient stratification.

The deconvolution analysis of tumor immune cells infiltration was performed using CIBERSORT, a computational approach able to estimate the immune composition starting from microarray or RNA-Seq data. The signature matrix includes 547 genes that accurately distinguish 22 mature human hematopoietic populations such as: T cell types, naïve and memory B cells, plasma cells, NK cells, and myeloid subsets^68^.

### Statistical analysis: radiogenomic correlation and clustering

Spearman’s rank correlation coefficient and clustering analysis were utilized to explore the associations between each radiomic and gene expression signature.

To elucidate relationships identified in the correlation analysis - specifically, whether certain radiomic signatures exhibit similar behavior in their relationships with gene expression signatures - a cluster-based approach was employed. Only stable clusters were considered, as determined through silhouette analysis^69,70^. Silhouette values, which range from − 1 to 1, were assigned to each observation in a dataset based on a set of cluster assignments. A higher silhouette value indicates better alignment with its own cluster and poorer alignment with neighboring clusters. The procedure involved several steps: (i) multiple clustering iterations (*n* = 50) using k-means clustering were conducted; (ii) silhouette values were computed for each data point in each clustering run; (iii) mean silhouette values for each data point were calculated across all runs; (iv) clusters with a mean silhouette value equal to or greater than the specified threshold, set to 0.2, were deemed stable^69,70^. This methodology facilitated the filtering of gene expression signatures to those most closely correlated with radiomic signatures, thus directing focus towards key relationships between radiomic signatures and immune status.

Finally, the correlation between radiomic signatures and immune cell populations was also examined to determine the potential connection of radiomics with the patient’s immune status.

The analysis was conducted using Matlab 2022a.

### Development and testing of predictive radiomic model of gene expression signatures

Radiomic classifiers were built for each of the correlation-filtered immune-related gene expression signatures that presented a minority class greater than 10% of the dataset, using the support vector machine method. Two analyses were performed: first, each radiomic signature was considered alone, and second, all radiomic signatures were combined to develop the radiomic model for gene expression prediction. For each analysis the workflow consisted in performing 100 stratified train-test splits (80%-20%) with different seeds of the random number generator to increase the robustness. At each of the 100 repetitions, (i) the minority class of the train dataset was oversampled through the synthetic minority oversampling technique (SMOTE) algorithm to obtain balanced classes^71^, (ii) the support vector machine classifier was trained on the train dataset and applied to the test dataset and (iii) the classifier performance metrics (e.g., balanced accuracy and AUC) were computed on the test set. Finally, the median and interquartile range of the balanced accuracy and AUC were computed over the 100 repetitions.

## Electronic supplementary material

Below is the link to the electronic supplementary material.

Supplementary Material 1

## Data Availability

All relevant data and materials have been included in the article and its supplementary data files. Further inquiries can be directed to the corresponding author.
