# Supplementary material for: Interplay between MRI radiomics and immune gene expression signatures in oral squamous cell carcinoma
Source: Sci Rep. 2025 Apr 12;15:12622. doi: 10.1038/s41598-025-96821-x (PMC11993570; doi:10.1038/s41598-025-96821-x)
Supplement: Supplementary file 1 — Supplementary Material 1 [file 41598_2025_96821_MOESM1_ESM.docx]

**Interplay between MRI radiomics and immune gene expression signatures in oral squamous cell carcinoma**

***- Supplementary Material -***

Anna Corti^1,*&^, Deborah Lenoci^2&^, Valentina D.A. Corino^1,3^, Davide Mattavelli^4^, Marco Ravanelli^5^, Tito Poli^6^, , Stefano Cavalieri^7,8^, Lisa Licitra^7,8^, Loris De Cecco^2#^, Luca Mainardi^1#^

1. Department of Electronics, Information and Bioengineering, Politecnico di Milano, Milan, Italy
2. Integrated Biology of Rare Tumors, Department of Research, Fondazione IRCCS, Istituto Nazionale dei Tumori, Milan, Italy
3. Cardiotech Lab, Centro Cardiologico Monzino IRCCS, Milan, Italy
4. Unit of Otorhinolaryngology-Head and Neck Surgery; Department of Medical and Surgical Specialties, Radiological Sciences, and Public Health; ASST Spedali Civili of Brescia, University of Brescia, Brescia, Italy
5. Unit of Radiology; Department of Medical and Surgical Specialties, Radiological Sciences, and Public Health; ASST Spedali Civili of Brescia, University of Brescia, Brescia, Italy
6. Maxillo-Facial Surgery Division, Head and Neck Department, University Hospital of Parma, Parma, Italy
7. Head and Neck Medical Oncology Department, Fondazione IRCCS Istituto Nazionale dei Tumori, Milan, Italy.
8. Department of Oncology and Hemato-Oncology, University of Milan, Milan, Italy.

***Address for correspondence:**

Anna Corti, PhD: Department of Electronics, Information and Bioengineering, Politecnico di Milano, Via Ponzio 34/5,20133 Milan, Italy - [anna.corti@polimi.it](mailto:anna.corti@polimi.it)

& co-first authors

# co-last authors

**Supplementary Table 1** Radiomic signatures with corresponding features.

| Radiomic signature | Features | Reference |
| --- | --- | --- |
| R1 | *T1wCont_waveletHLL_gldm_SmallDependenceEmphasis*  *T1wCont_waveletLLH_ngtdm_Busyness*  *T1wCont_waveletLLL_ngtdm_Busyness*  *T1wCont_waveletHHH_glszm_ZoneVariance*  *T1wCont_logsigma20mm3D_glcm_DifferenceVariance*  *T1wCont_waveletHHH_glszm_LargeAreaHighGrayLevelEmphasis*  *T1wCont_waveletHHH_ngtdm_Strength*  *T1wCont_waveletLHH_ngtdm_Complexity*  *T1wCont_waveletLHH_glcm_Correlation*  *T1wCont_logsigma20mm3D_glcm_InverseVariance* | Bos 2021 ^1^ |
| R2 | *T1wCont_original_shape_Maximum3DDiameter*  *T1wCont_original_shape_Compactness1*  *T1wCont_original_glrlm_RunLengthNonUniformityNormalized*  *T1wCont_waveletHLL_glrlm_LongRunEmphasis*  *T1wCont_waveletLHL_glcm_JointEntropy*  *T1wCont_waveletHLH_glrlm_ShortRunHighGrayLevelEmphasis* | Chen 2022 ^2^ |
| R3 | *T1w_original_glszm_SizeZoneNonUniformity*  *T1wCont_waveletLLL_ngtdm_Complexity*  *T2w_waveletHLL_gldm_DependenceVariance* | Alfieri 2022 ^3^ |
| R4 | *T1wCont_logsigma15mm3D_firstorder_90Percentile*  *T1wCont_logsigma10mm3D_firstorder_Energy*  *T1wCont_logsigma10mm3D_firstorder_TotalEnergy*  *T1wCont_waveletLHL_glszm_SizeZoneNonUniformity* | Siow 2022 ^4^ |
| R5 | *T2w_original_shape_Maximum2DDiameterRow*  *T2w_logsigma50mm3D_firstorder_Maximum* | Mossinelli 2023 ^5^ |
| R6 | *T1w_waveletLHL_firstorder_90Percentile*  *T2w_original_shape_VoxelVolume*  *T2w_waveletHHL_glrlm_GrayLevelNonUniformityNormalized*  *T2w_waveletLLL_firstorder_InterquartileRange*  *T2w_waveletLLL_firstorder_Range* | Bologna 2023 ^6^ |
| R7 | *T2w_waveletLLL_glrlm_LongRunEmphasis*  *T2w_waveletLLL_glrlm_RunVariance*  *T2w_waveletLLL_glrlm_RunPercentage*  *T2w_waveletLLL_firstorder_Range*  *T2w_waveletLLL_glrlm_ShortRunEmphasis* | Corti 2023 ^7^ |

**Supplementary Table 2** Gene expression signatures with corresponding genes.

| **Signature ID Hacksig** | **List of Immune Related genes** |
| --- | --- |
| G1  Li2020_immune related ^8^ | HPV-neg: PLAU, SFTPA2, CCL26, SEMA3G, DKK1, GAST, GNRH1, PDGFA, ZAP70, STC1 HPV-pos: SEMA3G, GNRH1, TNFRSF4, ZAP70, PLAU, SH2D1A, CCL26, DKK1, GAST, PDGFA, STC1 |
| G2  Fang2021_immune  Related ^9^ | immune-related genes: DEFB1, EDNRB, ADM, BTC, DKK1, FAM3D, GNRH1, STC2, TNFRSF12A ,CTLA4 |
| G3  Li2021_immune suppression ^10^ | Immune suppression genes (ISGs): BGLAP, CALCA, CTLA4, CXCL8, FGFR3, HPRT1, IL22, ORMDL3, TLR3, SPHK1, INHBB |
| G4  Lin2021_immune gene set ^11^ | stemness, immunity, and prognosis related genes ESCO2, CCNA2, COL5A3, RCN3, LMCD1, FMNL3, MMP14, HEYL |
| G5  Bai2019_immune inflammatory ^12^ | immune inflammatory genes CD27, CD79B, CMA1, CCR4, CCR7, CNR2, CTLA4, CTSG, GZMM, IL16, MASP1, SAA1, CCL11, TNFAIP3, BATF, IL19, PGLYRP4, and TREML1 |
| G6  Lv2022_hypoxia-immune ^13^ | Hypoxia and immune status associated genes: FAM122C, RNF157, RANBP17, SOWAHA, KIAA1211, RIPPLY2, INSL3, and DNAH1 |
| G7  Feng2021_stemness ^14^ | Stemness index associated genes: NPM3, H2AFZ, KPNA2, CCDC92, GAS1, CCL22, TSPAN11, CLEC3B, TWIST2, TPSAB1, IGLV2-14 |
| G8  *Dai2021_radio-sensitivity* ^15^ | Radiosensitivity-related genes: ACTN1, ANXA2, ANXA5, ARHGDIB, CAPNS1, CBR1, CCND1, CD63, CORO1A, CXCR4, DAG1, EMP2, HCLS1, HTRA1, ITGB5, LAPTM5, LRMP, MYB, PFN2, PIR, PKM, PTMS, PTPRC, PTPRCAP, PYGB, RAB13, RALB, SCRN1, SQSTM1, TWF1, WAS |
| G9  *Wang2021_immune related* ^16^ | prognostic genes in stromal/immune scores: RSPO1, CCR7, C3, MYL2, EVPLL, KHDC1L |
| G10  *Qiang2021_immune related* ^17^ | immune-related genes (IRGs): PLAU, IRF9, CCL26, BLNK, SEMA3G, FPR2, GAST, IL34, SLURP1, STC1, STC2, TNFRSF12A, TNFRSF25 |
| G11**  *Chen2022_regulator genes* ^18^ | m6A regulator-related genes: DAD1, CYCS, CSNK2A2, VPS25, VDAC1, TMLHE, CYTH3, PRKCA,TP73, FZD6, SQSTM1, MAP2K7 |
| G12  *Yang2020_immune related* ^19^ | Immune-related genes: LCN15, NOX5, PDGFA, PSMD6, PTX3, TMSB4Y |
| G13  *Long2020_immune related* ^20^ | Immune related genes associated to prognosis. PLAU, SFTPA2, PTX3, PDGFRB, CCL26, CD22, IGHV3-64, GAST, GNRH1, PDGFA, SLURP1, STC2, AR, TNFRSF25 |
| G14  *Liang2019_inflammation* ^21^ | Chemokines/receptors  Inflammation-Immunity: CCL22, XCL2, CCR4, CCR6, CCR7, XCR1, CX3CR1, CCL2, CCL7, CXCL5, CXCL8 |
| G15  *Chen2022_neutrophil trap* ^22^ | Neutrophil extracellular traps  (NET) related-genes: ANXA3, LTF, CSF2, GAPDH, CYBB, SELPLG |
| G16  *Li2022_ nerve cross-talk-related* ^23^ | nerve cross-talk-related genes (NCCGs): NTRK1, L1CAM, GRIN3A, CHRNA5, CHRNA6, CHRNB4, CHRND |
| G17  *Zhang2021_ m6A methylation-related* ^24^ | m6A methylation-related genes: YTHDC2, HNRNPA2B1, RBM15B |
| G18  *Ming2021_ DNA Repair Genes* ^25^ | DNA-repair-genes: MORF4L2 , COPS2, USP10, WAS, UVSSA, PRRX1, ZBTB1, DCLRE1C, MSH5, DOT1L, ZBTB7A, POLR2C, MORF4L1 |
| G19  *Zhang2022_immune features* ^26^ | lymph node metastasis associated genes: CXCL11, CXCR3, CCR5 |
| G20  *Yi2020_ RNA Methylation Regulators* ^27^ | m6 A RNA Methylation Regulators genes: YTHDC2, YTHDC1, YTHDF2, METTL14, RBM15, KIAA1429, ZC3H13, YTHDF3, FTO, HNRNPC, METTL3, WTAP, ALKBH5, METTL16, YTHDF1 |
| G21  *Feng2020_gene regulatory network* ^28^ | Gene prognostic for ICI therapy response: CTSW, CYFIP2, GZMM, ICAM2, IL12, IL17A, IL2, JAK3, KIR3DL3, XCL2 |
| G22  *Chen2021_immune related* ^29^ | immunerelated prognostic index (IRGPI): SFRP4, CPXM1, COL5A1 |
| G23  *Ming2022_immune related RNA-binding proteins* ^30^ | immune-related RBPs: FRMD4A, ASNS, RAB11FIP1, FAM120C, CFLAR, CTTN, PLEKHO1, SELENBP1, CHCHD2, NPM3, ATP2A3, CFDP1, IGF2BP2, NQO1, DENND2D |
| G24  *Chen2021_immune related* ^31^ | Immune related prognostic genes: IGSF5, NKX2-3, HLPF, ALDH2, IFIT2, FXYD5, CTSL1, IFNAR1, RNF216 |
| G25  *Lu2022_inflammation* ^32^ | Inflammation related genes: ADGRE1, OLR1, GPR132, TIMP1, CCR7, PSEN1, NAMPT |
| G26  *Cai2022_mast cell marker* ^33^ | Mast cell marker genes: KIT, RAB32, CATSPER1, SMYD3, LINC00996, SOCS1, AP2M1, LAT, HSP90B1 |
| G27  *Zhang2021_immune related* ^34^ | Immune-related genes: PLAU, STC2, TNFRSF4, PDGFA, DKK1, CHGB |
| G28  *Tang2022_immune cell infiltration* ^35^ | gene related to drug absorption, distribution, metabolism, excretion (ADME): UGT1A, SULT1B1, SOD1, SLCO1B3, SLCO1B1, PPARG, CYP8B1, CYP7B1, CYP4F12, CYP2D6, ALDH2, ABCB8 , ABCB4, ABCB1 |
| G29  *Zhang2022_CD8 infiltration* ^36^ | Immune related genes: DEFB1, AICDA, TYK2, CCR7, SCARB1, ULBP2, STC2, LGR5 |

**References**

1. Bos, P. *et al.* Improved outcome prediction of oropharyngeal cancer by combining clinical and MRI features in machine learning models. *Eur. J. Radiol.* **139**, 109701 (2021).

2. Chen, J. *et al.* An MRI-based radiomics-clinical nomogram for the overall survival prediction in patients with hypopharyngeal squamous cell carcinoma: a multi-cohort study. *Eur. Radiol.* **32**, 1548–1557 (2022).

3. Alfieri, S. *et al.* Prognostic role of pre-treatment magnetic resonance imaging (MRI)-based radiomic analysis in effectively cured head and neck squamous cell carcinoma (HNSCC) patients. *Acta Oncol.* **60**, 1192–1200 (2021).

4. Siow, T. Y. *et al.* MRI Radiomics for Predicting Survival in Patients with Locally Advanced Hypopharyngeal Cancer Treated with Concurrent Chemoradiotherapy. *Cancers (Basel).* **14**, 6119 (2022).

5. Mossinelli, C. *et al.* The role of radiomics in tongue cancer: A new tool for prognosis prediction. *Head Neck* **45**, 849–861 (2023).

6. Bologna, M. *et al.* Prognostic radiomic signature for head and neck cancer: Development and validation on a multi-centric MRI dataset. *Radiother. Oncol. J. Eur. Soc. Ther. Radiol. Oncol.* **183**, 109638 (2023).

7. Corti, A. *et al.* MRI-based radiomic prognostic signature for locally advanced oral cavity squamous cell carcinoma: development, testing and comparison with genomic prognostic signatures. *Biomark. Res.* **11**, 69 (2023).

8. Li, L. *et al.* Comprehensive immunogenomic landscape analysis of prognosis-related genes in head and neck cancer. *Sci. Rep.* **10**, 6395 (2020).

9. Fang, R. *et al.* A novel comprehensive immune-related gene signature as a promising survival predictor for the patients with head and neck squamous cell carcinoma. *Aging (Albany. NY).* **13**, 11507–11527 (2021).

10. Li, S. *et al.* Molecular Subtypes of Oral Squamous Cell Carcinoma Based on Immunosuppression Genes Using a Deep Learning Approach. *Front. cell Dev. Biol.* **9**, 687245 (2021).

11. Lin, X. *et al.* Clinical significance and immune landscapes of stemness-related and immune gene set-based signature in oral cancer. *Clinical and translational medicine* vol. 11 e343 at https://doi.org/10.1002/ctm2.343 (2021).

12. Bai, S. *et al.* Bioinformatic Analysis Reveals an Immune/Inflammatory-Related Risk Signature for Oral Cavity Squamous Cell Carcinoma. *J. Oncol.* **2019**, 3865279 (2019).

13. Lv, S., Qian, Z., Li, J., Piao, S. & Li, J. Identification and Validation of a Hypoxia-Immune-Based Prognostic mRNA Signature for Oral Squamous Cell Carcinoma. *J. Oncol.* **2022**, 5286251 (2022).

14. Feng, J., Li, Y. & Wen, N. Characterization of Cancer Stem Cell Characteristics and Development of a Prognostic Stemness Index Cell-Related Signature in Oral Squamous Cell Carcinoma. *Dis. Markers* **2021**, 1571421 (2021).

15. Dai, D. *et al.* Combination of Radiosensitivity Gene Signature and PD-L1 Status Predicts Clinical Outcome of Patients With Locally Advanced Head and Neck Squamous Cell Carcinoma: A Study Based on The Cancer Genome Atlas Dataset. *Front. Mol. Biosci.* **8**, 775562 (2021).

16. Wang, Z. *et al.* Prognostic value of immune-related genes and immune cell infiltration analysis in the tumor microenvironment of head and neck squamous cell carcinoma. *Head Neck* **43**, 182–197 (2021).

17. Qiang, W., Dai, Y., Xing, X. & Sun, X. Identification and validation of a prognostic signature and combination drug therapy for immunotherapy of head and neck squamous cell carcinoma. *Comput. Struct. Biotechnol. J.* **19**, 1263–1276 (2021).

18. Chen, J. *et al.* A Signature of N(6)-methyladenosine Regulator-Related Genes Predicts Prognoses and Immune Responses for Head and Neck Squamous Cell Carcinoma. *Front. Immunol.* **13**, 809872 (2022).

19. Yang, J., Xie, K. & Li, C. Immune-related genes have prognostic significance in head and neck squamous cell carcinoma. *Life Sci.* **256**, 117906 (2020).

20. Long, J. *et al.* Development of an Immunogenomic Landscape-Based Prognostic Index of Head and Neck Squamous Cell Carcinoma. *Front. Mol. Biosci.* **7**, 586344 (2020).

21. Liang, Y. *et al.* An Inflammation-Immunity Classifier of 11 Chemokines for Prediction of Overall Survival in Head and Neck Squamous Cell Carcinoma. *Med. Sci. Monit. Int. Med. J. Exp. Clin. Res.* **25**, 4485–4494 (2019).

22. Chen, N., He, D. & Cui, J. A Neutrophil Extracellular Traps Signature Predicts the Clinical Outcomes and Immunotherapy Response in Head and Neck Squamous Cell Carcinoma. *Front. Mol. Biosci.* **9**, 833771 (2022).

23. Li, J. *et al.* Identification of the Nerve-Cancer Cross-Talk-Related Prognostic Gene Model in Head and Neck Squamous Cell Carcinoma. *Front. Oncol.* **11**, 788671 (2021).

24. Zhang, Y. *et al.* Identification of m6A methyltransferase-related genes predicts prognosis and immune infiltrates in head and neck squamous cell carcinoma. *Ann. Transl. Med.* **9**, 1554 (2021).

25. Ming, R. *et al.* The Prognostic Value of the DNA Repair Gene Signature in Head and Neck Squamous Cell Carcinoma. *Front. Oncol.* **11**, 710694 (2021).

26. Zhang, Y., Luo, X., Yu, J., Qian, K. & Zhu, H. An Immune Feature-Based, Three-Gene Scoring System for Prognostic Prediction of Head-and-Neck Squamous Cell Carcinoma. *Front. Oncol.* **11**, 739182 (2021).

27. Yi, L., Wu, G., Guo, L., Zou, X. & Huang, P. Comprehensive Analysis of the PD-L1 and Immune Infiltrates of m(6)A RNA Methylation Regulators in Head and Neck Squamous Cell Carcinoma. *Mol. Ther. Nucleic Acids* **21**, 299–314 (2020).

28. Feng, B. *et al.* Integrative Analysis of Multi-omics Data Identified EGFR and PTGS2 as Key Nodes in a Gene Regulatory Network Related to Immune Phenotypes in Head and Neck Cancer. *Clin. cancer Res. an Off. J. Am. Assoc. Cancer Res.* **26**, 3616–3628 (2020).

29. Chen, Y., Li, Z.-Y., Zhou, G.-Q. & Sun, Y. An Immune-Related Gene Prognostic Index for Head and Neck Squamous Cell Carcinoma. *Clin. cancer Res. an Off. J. Am. Assoc. Cancer Res.* **27**, 330–341 (2021).

30. Ming, R. *et al.* The Prognostic Signature of Head and Neck Squamous Cell Carcinoma Constructed by Immune-Related RNA-Binding Proteins. *Front. Oncol.* **12**, 795781 (2022).

31. Chen, Y. *et al.* Identification of Immune-Related Prognostic Biomarkers Associated with HPV-Positive Head and Neck Squamous Cell Carcinoma. *J. Immunol. Res.* **2021**, 6661625 (2021).

32. Lu, Y. & Jia, Z. Inflammation-Related Gene Signature for Predicting the Prognosis of Head and Neck Squamous Cell Carcinoma. *Int. J. Gen. Med.* **15**, 4793–4805 (2022).

33. Cai, Z., Tang, B., Chen, L. & Lei, W. Mast cell marker gene signature in head and neck squamous cell carcinoma. *BMC Cancer* **22**, 577 (2022).

34. Zhang, Y. *et al.* A Novel Immune-Related Prognostic Signature in Head and Neck Squamous Cell Carcinoma. *Front. Genet.* **12**, 570336 (2021).

35. Tang, X. *et al.* Development and Validation of an ADME-Related Gene Signature for Survival, Treatment Outcome and Immune Cell Infiltration in Head and Neck Squamous Cell Carcinoma. *Front. Immunol.* **13**, 905635 (2022).

36. Zhang, S., Zhang, W. & Zhang, J. 8-Gene signature related to CD8(+) T cell infiltration by integrating single-cell and bulk RNA-sequencing in head and neck squamous cell carcinoma. *Front. Genet.* **13**, 938611 (2022).
